# How image quality affects determination of target displacement when using kilovoltage cone‐beam computed tomography

**DOI:** 10.1120/jacmp.v8i1.2440

**Published:** 2007-02-28

**Authors:** Seungjong Oh, Siyong Kim, Tae‐Suk Suh

**Affiliations:** ^1^ Department of Biomedical Engineering The Catholic University of Korea, College of Medicine Seoul Republic of Korea; ^2^ Department of Radiation Oncology Mayo Clinic Jacksonville Florida

**Keywords:** IGRT, reconstruction resolution, target displacement, cone‐beam CT

## Abstract

The advent of kilovoltage cone‐beam computed tomography (CBCT) integrated with a linear accelerator allows for more accurate image‐guided radiotherapy (IGRT). The IGRT technique corrects target displacement based on internal body information obtained by acquiring the CBCT image set and registering it with the simulation CT image set just before the beam is delivered. In the present paper, we compare registration results by CBCT reconstruction quality (either high or medium). We analyzed data from a total of 56 CBCT projections from 6 patients. The translational vector differences were within 1 mm in all but 3 cases. The rotational displacement differences considered components of all three axes, and in 3 of 168 cases (56 projections by 3 axes), showed more than 1 degree of difference.

PACS number: 87.57.Nk

## I. INTRODUCTION

Imperfection in treatment setup is one of the main causes of interfraction target displacement. The process of treatment setup conventionally uses skin marks and room laser systems, with setup accuracy then depending heavily on information from the patient's external body rather than the patient's internal organs. The lack of information about internal organs requires that a significant planning margin be added to the target area to compensate for inaccuracies of target localization in conventional patient setup—especially for regions subject to severe motion over time, such as liver or lungs.

Recent advances in treatment technique, such as intensity‐modulated radiation therapy and stereotactic body radiation therapy, have increased the demand for more accurate target localization. Kilovoltage cone‐beam computerized tomography (CBCT) is one method used to localize the target volume based on information about internal organs. Reports indicate that setup error can successfully be corrected by adjusting translational and rotational deviations between the planning computed tomography (CT) image (the reference image) and the CBCT images taken before beam delivery.(^1^–^3^)

Using the Elekta Synergy (Elekta Oncology Systems, Norcross, GA) linear accelerator (LINAC), CBCT images can be reconstructed from the projection data at three quality levels: low, medium, and high. High‐quality reconstruction provides images in a resolution higher than that of the medium‐ and low‐quality reconstructions—for example, in the medium field size, the resolutions of high‐, medium‐, and low‐quality reconstructions are 1024, 512, and 250 pixels respectively (Table 1). However, a longer time is needed to create a high‐quality image reconstruction—about 3.5 minutes, versus about 1.5 minutes for medium‐ and low‐quality reconstructions. The difference in time between medium‐ and low‐quality image reconstructions is not significant because CBCT reconstruction begins right after gantry rotation starts, and most of the process can be finished during rotation, which generally takes more than a minute. Medium‐quality reconstruction therefore makes efficient use of CBCT, if the image quality does not significantly compromise target localization accuracy.

**Table 1:** Image resolution of Elekta XVI in terms of field of view (FOV) and reconstruction quality

	Reconstruction quality (mm/pixel)		
FOV	Low (250 pixels)	Medium (512 pixels)	High (1024 pixels)
Small (260mm)	1.02	0.51	0.25
Medium (400mm)	1.56	0.78	0.39
Large (600mm)	2.34	1.17	0.59

In the present paper, we report our investigation of the effect of image quality on target localization from a number of clinical cases.

## II. Methods

### A. Equipment

We used the Elekta Synergy LINAC to obtain CBCT projection data. Image reconstruction from the projection data and image registration were performed using the iViewGT (Elekta Oncology Systems, Crawley, UK) portal image system.

### B. Data acquisition and image registration

**Figure 1:**
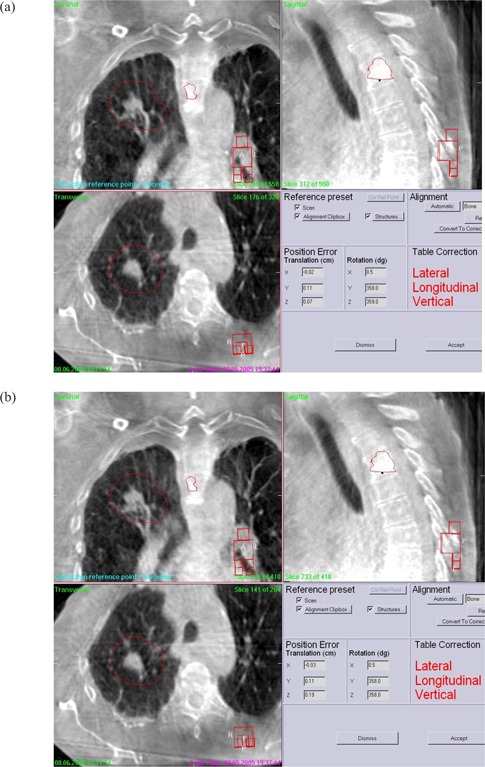
(a) High‐ and (b) medium‐quality reconstructed cone‐beam computed tomography images of lung and spine area. Both images clearly show lung mass and spinal bones. No easily observable significant difference is seen between the reconstructions.

We obtained data from a total of 56 CBCT projections during treatment of three disease sites (spine, lung, and prostate) in 6 patients treated with a kV CBCT–integrated Elekta Synergy LINAC. All data were acquired in the medium field of view (20×27 cm). For each projection data set, images were reconstructed at both high and medium quality (Fig. 1). The pixel sizes of the reconstructed images were 0.78 mm for medium resolution and 0.39 mm for high resolution. The reconstructed CBCT images were registered to a reference planning CT image to obtain target displacements.

The registration was performed in this order:

- The default region of interest (ROI) for each patient was defined on the reference planning CT.
- Any significant translational discrepancy of the internal anatomy between the reference planning CT image and the CBCT image was roughly adjusted in manual mode.
- The ROI was redefined with fine tuning if necessary.
- The two images were matched based on bony anatomy.
- The registered images were checked and accepted.

**Figure 2:**
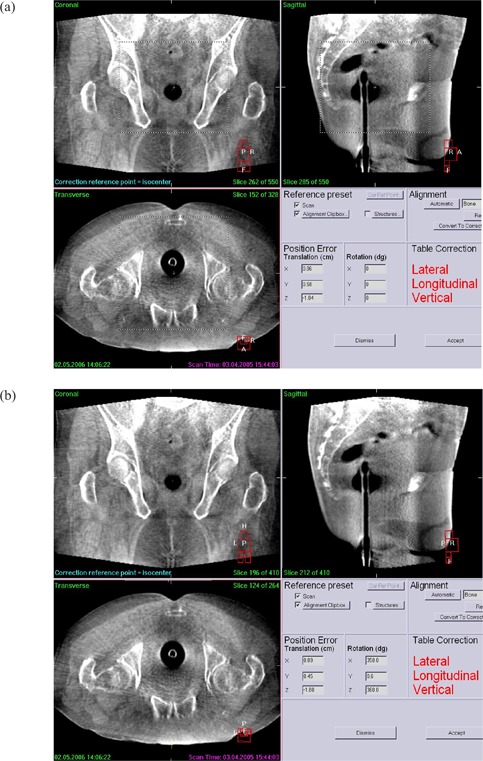
(a) High‐ and (b) medium‐quality reconstructed cone‐beam computed tomography images of the prostate area. The area enclosed by the dotted box is a typical region of interest used for image registration with reference to a computed tomography scan. As with the lung and spinal region images, no easily observable significant difference is seen between the reconstructions.

The automated registration algorithm based on bony anatomy was used because the manual rotation correction was difficult, and the registration result was not acceptable. After the registered image had been accepted, the translational and rotational displacements were obtained. The accuracy of the medium‐quality CBCT image was evaluated by comparing the displacement results between the medium‐quality image and the corresponding high‐quality image, because setup error was corrected clinically from high‐quality CBCT images (Fig. 2).

## III. RESULTS

**Figure 3:**
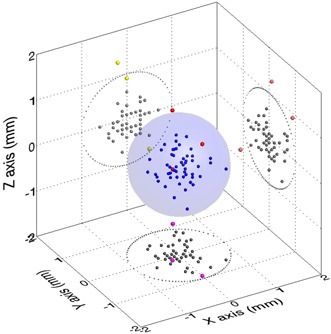
Translational vector differences of determined target displacements using the high‐ and medium‐quality resolution cone‐beam computed tomography images. The points in three‐dimensional (3D) space are projected onto each plane. The red spheres indicate 3D translation vector differences in excess of 1 mm. Violet, orange, and yellow spheres represent projection of those vector differences onto each plane (xy, yz, and zx respectively).

Of the 56 CBCT projections analyzed (19 from lung, 20 from prostate, and 17 from spine), the translational vector differences between the registration results of high‐and medium‐quality CBCT images were less than 1 mm, except for 3 cases: 1 from spine (1.45 mm) and 2 from lung (1.27 mm and 1.32 mm; Fig. 3). In Fig. 3, points in the three‐dimensional (3D) space are also projected onto each plane. The red spheres indicate points with more than a 1‐mm translational vector difference in 3D space. Violet, orange, and yellow spheres represent the projection onto each plane (xy, yz, and zx respectively), where the x‐axis represents the right–left direction; the y‐axis, the superior–inferior direction; and the z‐axis, the anterior–posterior direction.

For rotational displacements, components of all three axes were considered, and of 168 cases (56 projections by 3 axes), just 3 showed more than 1 degree of difference: one around the x‐axis (1.7 degrees, lung), one around the y‐axis (1.5 degrees, spine), and one around the z‐axis (1.1 degree, lung). The first and third cases correspond to 2 of the 3 cases that showed more than 1 mm of translational vector differences.

The means and standard deviations of the translational differences were 10×10 mm along the x‐axis, 0.30 ± 0.27 mm along the y‐axis, and 0.26 ± 0.25 mm along the z‐axis. The vector mean and standard deviation was 0.53 ± 0.30 mm. For rotational differences, the means and standard deviations were 0.47 ± 0.51 degrees around the x‐axis, 0.58 ± 1.36 degrees around the y‐axis, and 0.4 ± 0.51 degrees around the z‐axis. Table 2 summarizes the results.

**Table 2:** Differences in target translational and rotational displacements, as determined by (a) high‐ and (b) medium‐quality cone‐beam computed tomography image sets; results are represented by average, standard deviation, and maximum (max) value

Organ	Patient	Data set (*N*)	$t_{x}$ (max)	Absolute translational difference (mm) $t_{y}$ (max)	$t_{z}$ (max)	Absolute translational vector difference (mm)	$r_{x}^{{}^{\circ}}$ (max)	Absolute rotational difference (degrees) $r_{y}^{{}^{\circ}}$ (max)	$r_{z}^{{}^{\circ}}$ (max)
Lung	(a)	9	0.32±0.17 (0.5)	0.37±0.26 (0.8)	0.17±0.33 (0.5)	0.56±0.28 (0.99)	0.33±0.33 (1)	0.37±0.48 (1)	0.47±0.51 (1)
	(b)	10	0.27±0.19 (0.7)	0.28±0.36 (0.9)	0.50±0.41 (1.3)	0.76±0.36 (1.32)	0.34±0.17 (0.6)	0.61±0.53 (1.5)	0.38±0.50 (1.1)
Prostate	(a)	10	0.13±0.12 (0.4)	0.32±0.20 (0.7)	0.27±0.14 (0.4)	0.48±0.19 (0.74)	0.44±0.53 (1)	0.12±0.16 (0.5)	0.11±0.14 (1)
	(b)	10	0.18±0.11 (0.3)	0.32±0.28 (0.7)	0.26±0.24 (0.7)	0.51±0.28 (0.87)	0.56±0.53 (1)	0.28±0.30 (1)	0.26±0.43 (1)
Spine	(a)	10	0.14±0.09 (0.6)	0.13±0.15 (0.5)	0.16±0.13 (0.4)	0.30±0.13 (0.63)	0.26±0.38 (1)	0.39±0.47 (1)	0.30±0.13 (0.9)
	(b)	7	0.26±0.14 (0.5)	0.43±0.37 (1.1)	0.21±0.27 (0.8)	0.59±0.41 (1.45)	0.61±0.65 (1.7)	0.57±0.42 (1)	0.51±0.46 (1)
Total		56	0.23±0.16	0.30±0.27	0.26±0.25	0.53±0.30	0.47±0.51	0.58±1.36	0.40±0.51

## IV. DISCUSSION

In comparing the estimated target displacements obtained using two CBCT image sets (one in high‐quality resolution and the other in medium‐quality resolution), we found that the translational vector differences between the high‐quality resolution and medium‐quality resolution were within 1 mm in most cases (53 of 56 cases), and that the rotational differences around each axis were within 1 degree in all but 3 cases.

Oldham et al. reported that the accuracy of CBCT‐guided radiation therapy was within 1 pixel and that the intra‐user registration error was about 0.5 mm.^(4)^ Also, Sharpeb et al. reported that the mechanical accuracy and reproducibility of CBCT were about 1 mm.^(5)^ Considering those reports, the mean and standard deviation of translational vector differences found in our study (0.53±0.30 mm) are acceptable. Studies of rotational displacement like the present one are rare, because corrections in rotational displacement are not made in most cases; the hardware is not capable of adjusting rotation.

Generally, an increase in treatment time means an increase in patient motion. If the reconstruction time is long, patient motion may cause the target to migrate from the position it occupied at the time that the CBCT projection data was acquired. Although a high‐quality image offers more detailed information, no severe discrepancy of setup error correction is observed when a medium‐quality image is used instead.

## ACKNOWLEDGMENTS

The present work was financially supported by the Seoul R&BD Program (10550) and the Mid‐ and Long‐Term Nuclear R&D Program of the Ministry of Science and Technology (M20513000010‐06A1300‐01010).
